# Mediastinal Cystic Lymphangioma in a Patient with Situs Inversus Totalis

**DOI:** 10.1155/2014/781874

**Published:** 2014-11-06

**Authors:** Teruya Komatsu, Yutaka Takahashi

**Affiliations:** ^1^Department of General Thoracic Surgery, Kyoto University Hospital, 54 Kawaharacho, Shogoin, Sakyo-ku, Kyoto 606-8507, Japan; ^2^Department of General Thoracic Surgery, Kobe City Medical Center General Hospital, 1-1, 2 Tyoume, Minatozima-minamimachi, Tyuou-ku, Kobe, Hyogo 650-0047, Japan

## Abstract

We present a case of cystic lymphangioma of the mediastinum complicated with situs inversus totalis. The 70-year-old man underwent thoracoscopic resection of a mediastinal cystic tumor, which was diagnosed as cystic lymphangioma. Cystic lymphangiomas are congenital cystic abnormalities of the lymphatic system. The head and neck area is often involved while the mediastinum is rarely affected. The rarity of this case is further attributed to the coexistence of situs inversus totalis.

## 1. Introduction

Cystic lymphangioma is a rare congenital anomaly of the lymph system, usually detected within 2 years after birth [1]. The most commonly affected sites are the cervical and axillary areas. Mediastinal cystic lymphangioma is rare, accounting for 0.7% to 4.5% of all mediastinal tumors [2]. To our knowledge, this is the first report of a case of mediastinal cystic lymphangioma with coexistence of situs inversus totalis.

## 2. Case Report

**Figure 1:**
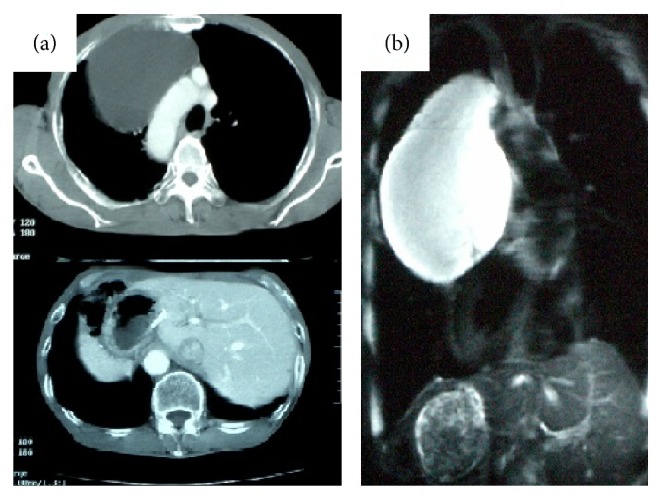
(a) Enhanced computed tomography scan of the whole body shows a right mediastinal cystic mass with a right-sided aortic arch and liver, consistent with situs inversus totalis. (b) T2-weighted magnetic resonance imaging scan of the chest shows a homogenous, high-intensity area adjacent to the right side of the mediastinum.

**Figure 2:**
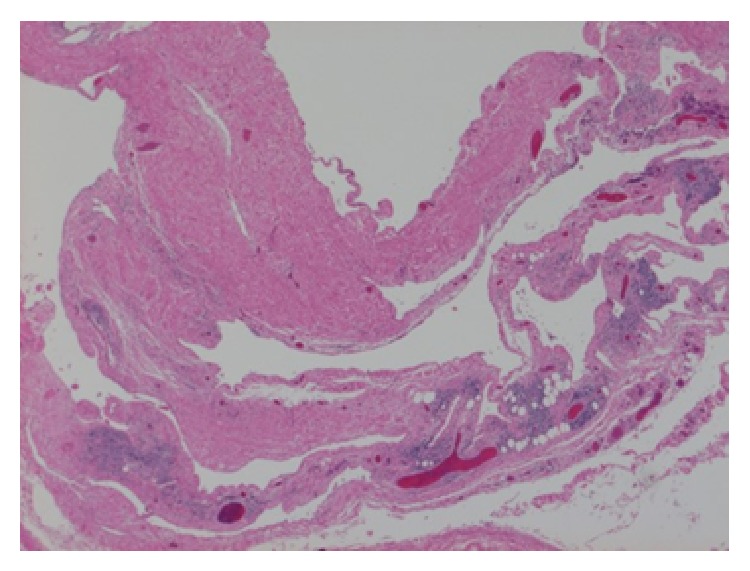
Histopathological examination (hematoxylin and eosin staining, original magnification ×100) of the cystic tumor showed lymphoid tissue and flattened squamous cells in the cystic wall, which was consistent with the findings of cystic lymphangioma.

A 70-year-old man was referred to our hospital for further investigation of a large mediastinal mass. This was detected on chest radiography performed for the assessment of dyspnea on exertion. The patient's past medical history included a subtotal gastrectomy for gastric cancer at the age of 60, with no other reported comorbidities. The previous chest X-ray for the preoperative workup of gastric cancer was not available; however, the perioperative course for the gastric cancer had been uneventful. Chest and abdominal computed tomography (CT) on referral showed the well demarcated mass of 80 mm in diameter, which was abutting the surrounding structures (the right chest wall, bilateral visceral pleura, and aortic arch) and situs inversus totalis. The internal homogenously low attenuation area was consistent with a cystic tumor (Figure 1(a)). The cystic nature was reconfirmed with magnetic resonance image (MRI) which showed the high intensity area of T2 weighted images (Figure 1(b)). Any other tumorous lesion was not found radiologically. Considering the radiological characteristics of the tumor, it had been strongly suspected to be cystic, and a less invasive procedure like transthoracic or bronchoscopic biopsy was supposed less likely to yield definitive diagnosis. Therefore, for therapeutic as well as diagnostic purposes, video-assisted thoracoscopic surgery was performed. The large size of the cystic tumor resulted in the obstruction of the operative field. In order to decrease its size, we performed aspiration of the cystic fluid which was serous and amounted to approximately 200 mL, and subsequently the cystic capsule was dissected and removed. The situs inversus totalis did not obstruct the thoracoscopic procedure. The specimen was confirmed to be a cystic lymphangioma on the basis of the histopathology report (Figure 2). The patient's postoperative recovery was uneventful, and the postoperative follow-up was performed for 2 years without any relapse.

## 3. Discussion

Lymphangiomas are regarded as a congenital anomaly arising from the lymphatic systems [3]. Most patients with mediastinal cystic lymphangioma are asymptomatic, although chest tightness and dyspnea can be reported, similar to our case [4]. According to previous reports [1, 3–5], mediastinal cystic lymphangioma is a rare entity, typically located in the right superior mediastinum. Brown and colleagues reported that mediastinal cystic lymphangioma accounted for 1.8% of all mediastinal cysts over a 40-year period [3]. In our experience, the radiographic appearance is distinct, but it is difficult to establish a diagnosis of cystic lymphangioma radiologically. A chest CT scan usually reveals a well-circumscribed fluid density lesion. This appears as a homogeneous high-intensity area on a T2-weighted MRI scan, findings consistent with a cyst. The differential diagnosis includes bronchogenic cysts, thymomas, and thymic cysts [1]. Surgical resection, via thoracotomy or thoracoscopy, provides a definitive diagnosis and is the standard option for treatment and a definitive diagnosis. Malignant transformation has not been reported.

In our case, the definitive diagnosis was made on the basis of hematoxylin and eosin staining, but, as Hunt and colleagues propose in their report, immunohistochemical staining for CD31 might be helpful [1].

Situs inversus totalis is an uncommon entity with an incidence in the range of 1 : 10 000 to 1 : 20 000 [6]. There is no evidence that the presence of situs inversus predisposes one to the development of lymphangioma. Technical difficulties regarding laparoscopic cholecystectomy for patients with situs inversus totalis have been acknowledged [6], but, in our experience, thoracoscopic resection was accomplished without difficulties.

## 4. Conclusion

Our case report illustrates the exceptionally rare case of cystic lymphangioma, complicated with situs inversus totalis. Surgical excision, either via thoracoscopy or via thoracotomy, is proposed for not only diagnostic but also therapeutic purposes.
